# Local Optogenetic Induction of Fast (20–40 Hz) Pyramidal-Interneuron Network Oscillations in the *In Vitro* and *In Vivo* CA1 Hippocampus: Modulation by CRF and Enforcement of Perirhinal Theta Activity

**DOI:** 10.3389/fncel.2016.00108

**Published:** 2016-04-26

**Authors:** Julien Dine, Andreas Genewsky, Florian Hladky, Carsten T. Wotjak, Jan M. Deussing, Walter Zieglgänsberger, Alon Chen, Matthias Eder

**Affiliations:** ^1^Max Planck Institute of PsychiatryMunich, Germany; ^2^Department “Stress Neurobiology and Neurogenetics”, Max Planck Institute of PsychiatryMunich, Germany; ^3^Scientific Core Unit “Electrophysiology and Neuronal Network Dynamics”, Max Planck Institute of PsychiatryMunich, Germany; ^4^Research Group “Neuronal Plasticity”, Max Planck Institute of PsychiatryMunich, Germany; ^5^Research Group “Molecular Neurogenetics”, Max Planck Institute of PsychiatryMunich, Germany; ^6^The Ruhman Family Laboratory for Research on the Neurobiology of Stress, Department of Neurobiology, Weizmann Institute of ScienceRehovot, Israel

**Keywords:** CA1, CRF, gamma, hippocampus, optogenetics, perirhinal cortex, pyramidal-interneuron network oscillations, theta

## Abstract

The neurophysiological processes that can cause theta-to-gamma frequency range (4–80 Hz) network oscillations in the rhinal cortical-hippocampal system and the potential connectivity-based interactions of such forebrain rhythms are a topic of intensive investigation. Here, using selective Channelrhodopsin-2 (ChR2) expression in mouse forebrain glutamatergic cells, we were able to locally, temporally precisely, and reliably induce fast (20–40 Hz) field potential oscillations in hippocampal area CA1 *in vitro* (at 25°C) and *in vivo* (i.e., slightly anesthetized NEX-Cre-ChR2 mice). As revealed by pharmacological analyses and patch-clamp recordings from pyramidal cells and GABAergic interneurons *in vitro*, these light-triggered oscillations can exclusively arise from sustained suprathreshold depolarization (~200 ms or longer) and feedback inhibition of CA1 pyramidal neurons, as being mandatory for prototypic pyramidal-interneuron network (P-I) oscillations. Consistently, the oscillations comprised rhythmically occurring population spikes (generated by pyramidal cells) and their frequency increased with increasing spectral power. We further demonstrate that the optogenetically driven CA1 oscillations, which remain stable over repeated evocations, are impaired by the stress hormone corticotropin-releasing factor (CRF, 125 nM) *in vitro* and, even more remarkably, found that they are accompanied by concurrent states of enforced theta activity in the memory-associated perirhinal cortex (PrC) *in vivo*. The latter phenomenon most likely derives from neurotransmission via a known, but poorly studied excitatory CA1→PrC pathway. Collectively, our data provide evidence for the existence of a prototypic (CRF-sensitive) P-I gamma rhythm generator in area CA1 and suggest that CA1 P-I oscillations can rapidly up-regulate theta activity strength in hippocampus-innervated rhinal networks, at least in the PrC.

## Introduction

There is overwhelming evidence that several cognitive functions in mammals require theta (4–12 Hz) and/or gamma (30–80 Hz) network oscillations in the hippocampus and associated rhinal cortices, including the perirhinal cortex (PrC; Zola-Morgan et al., 1993; Bilkey and Heinemann, 1999; Fell et al., 2001; Colgin and Moser, 2010; Buzsáki and Wang, 2012). The potential generation mechanisms and connectivity-based interactions of such forebrain rhythms are, however, still a matter of intense research. Gamma oscillations in the CA1 output subfield of the hippocampus, which presumably play an important role in working memory (Montgomery and Buzsáki, 2007), are widely thought to depend on oscillatory drive from area CA3 or the entorhinal cortex (Colgin and Moser, 2010). Yet, there exist also studies indicating that the CA1 network can intrinsically generate gamma oscillations (Whittington et al., 1995, 1997; Pietersen et al., 2014; Schomburg et al., 2014; Craig and McBain, 2015). Two experimentally induced types of this self-produced rhythmicity (and most likely also natural CA1 gamma oscillations) involve feedback inhibition of pyramidal neurons (Whittington et al., 1997; Buzsáki and Wang, 2012; Pietersen et al., 2014), but it remains unclear whether area CA1 harbors the circuitry for a generation of prototypic pyramidal-interneuron network (P-I) gamma oscillations. Such oscillations must exclusively arise from sustained suprathreshold depolarization and feedback inhibition of pyramidal cells (Bartos et al., 2007; Tiesinga and Sejnowski, 2009; ter Wal and Sejnowski, 2014), without direct excitatory effects of the induction agents/techniques on the mediating GABAergic interneurons (cf., Whittington et al., 1997; Pietersen et al., 2014; Yi et al., 2014), and could impact network oscillations in the PrC (Bilkey and Heinemann, 1999; Fell et al., 2001). Rationale for this assumption is given by the facts that area CA1 and the directly interconnected subiculum send excitatory projections to the PrC (van Groen and Wyss, 1990) and that pyramidal cells persistently fire in a synchronized rhythmic manner during P-I oscillations (Tiesinga and Sejnowski, 2009; ter Wal and Sejnowski, 2014). In the present study, we tested these scenarios in NEX-Cre-Channelrhodopsin-2 (ChR2) mice, which selectively express the excitatory opsin ChR2 in forebrain glutamatergic cells (and thus in CA1 pyramidal neurons), but not in inhibitory interneurons (Goebbels et al., 2006; Section “Results”). Similar optogenetic strategies have been previously used to investigate the role of fast-spiking interneurons in neocortical gamma oscillations (Cardin et al., 2009; Sohal et al., 2009), to induce gamma band activity in the somatosensory cortex and CA3 region (Adesnik and Scanziani, 2010; Akam et al., 2012), and to trigger ripple (>90 Hz) oscillations in area CA1 (Stark et al., 2014). In particular, we performed different types of electrophysiological measurements in acute brain slices and slightly anesthetized animals and combined them with sustained local ChR2 activation in dorsal area CA1.

## Materials and Methods

### Animals

All experimental procedures were approved by the committee for the Care and Use of Laboratory animals of the Government of Upper Bavaria, Germany. Mice selectively expressing ChR2(H134R)-EYFP in forebrain glutamatergic neurons were generated by breeding homozygous NEX-Cre mice (Goebbels et al., 2006) to homozygous Ai32 mice (Madisen et al., 2012; purchased from the Jackson Laboratory). Genotyping was performed using the following primers specific for NEX-Cre: NEXCre4 5′-GAG-TCC-TGG-AAT-CAG-TCT-TTT-TC-3′, NEXCre5 5′-AGA-ATG-TGG-AGT-AGG-GTG-AC-3′, and NEXCre6 5′-CCG-CAT-AAC-CAG-TGA-AAC-AG-3′. Standard PCR conditions resulted in a Cre-specific PCR product of 525-bp and a wild-type PCR product of 770-bp. Genotyping for ChR2 was conducted according to the genotyping protocol provided by the Jackson Laboratory. All animals were housed under a 12 h light/dark cycle starting 5 days before the experiment and had access to water and food *ad libitum*. All experiments were performed in 8- to 12-week-old male mice.

### Preparation of Brain Slices

Mice were anesthetized with isoflurane and decapitated. All following steps were done in ice-cold cutting saline saturated with carbogen gas (95% O_2_/5% CO_2_). This saline (pH 7.4) consisted of (in mM): 125 NaCl, 2.5 KCl, 25 NaHCO_3_, 1.25 NaH_2_PO_4_, 0.5 CaCl_2_, 6 MgCl_2_, and 25 D-glucose. After decapitation, the brain was rapidly removed from the cranial cavity and 350-μm-thick coronal slices containing the hippocampus were cut using a vibratome (HM650V; Thermo Scientific). Afterwards, slices were incubated for 30 min in carbogenated physiological saline at 34°C. This saline (pH 7.4) consisted of (in mM): 125 NaCl, 2.5 KCl, 25 NaHCO_3_, 1.25 NaH_2_PO_4_, 2 CaCl_2_, 1 MgCl_2_, and 25 D-glucose. Subsequently, slices were stored at room temperature (25°C) for at least 30 or 90 min in carbogenated physiological saline before patch-clamp or local field potential (LFP) recordings, respectively.

### Electrophysiology and Light Application *In Vitro*

All experiments were carried out at 25°C. In the submerged-type recording chamber, slices were continuously superfused with carbogenated physiological saline (5–6 ml/min flow rate according to Lu et al., 2012). Field potentials in the CA1 stratum pyramidale were recorded using glass microelectrodes (~1 MΩ open-tip resistance, filled with physiological saline) that were connected to an extracellular amplifier (EXT-01, npi electronic). Recording data were low-pass filtered at 500 Hz and digitized at 2.5 kHz.

For patch-clamp recordings, individual neurons in area CA1 were visualized by infrared videomicroscopy and the gradient contrast system. Somatic whole-cell patch-clamp recordings from CA1 pyramidal cells and interneurons (>1 GΩ seal resistance) were performed in bridge or voltage-clamp mode (−70 mV holding potential if not stated otherwise) using a SEC-10L intracellular amplifier (npi electronic). The potential/current was low-pass filtered at 1.3 kHz and digitized at 6.5 kHz. The patch-clamp electrodes (5–7 MΩ open-tip resistance) were pulled from borosilicate glass capillaries and, if not stated otherwise, filled with a solution consisting of (in mM): 130 K-gluconate, 5 NaCl, 2 MgCl_2_, 0.5 EGTA, 10 HEPES, 2 Mg-ATP, 0.3 Na-GTP, 20 phosphocreatine, and 5 D-glucose (potentials were corrected for a liquid junction potential of 12 mV). In some experiments, a high Cl^−^ intracellular solution was used. This solution contained (in mM): 140 KCl, 5 NaCl, 0.1 EGTA, 10 HEPES, 2 Mg-ATP, 0.3 Na-GTP, and 20 phosphocreatine (potentials were corrected for a liquid junction potential of 6 mV). If utilized, QX-314 was added at 2 mM to the intracellular solution. The access resistance (*R*_a_) was continuously monitored. Recordings were terminated if *R*_a_ changed >10%.

ChR2 was activated by a stabilized 100 W halogen light source (Hal 100, Zeiss; for a more detailed description see Section “Results”) or a Sapphire 488 nm laser (75 mW max. output power, Coherent). The light beam of the laser was collimated into an optical fiber (BFL37-200, Thorlabs), which was coupled into the epifluorescence port of an Axioskop 2 FS microscope (Zeiss; Dine et al., 2014). The duration of the light pulses was regulated by means of a LS3ZM2 shutter and VCM-D1 shutter driver (Vincent Associates). Light intensities in the focus plane were measured using the PM100 system (Thorlabs).

### Electrophysiology, Light Application, and Histology *In Vivo*

Mice were anesthetized with isoflurane in air (induction 4%, surgery 2.5%, recording 1.2%), while the body temperature was maintained at 36°C with a homeothermic blanket. Analgesic medication (Metacam, i.p.; lidocaine, locally) was administered before surgery. Mice were placed and secured in a stereotaxic frame and unilaterally implanted in the dorsal CA1 region (coordinates from bregma: AP −2.3 mm, ML +1.65 mm, DV 1.5 mm) with an optrode, which consisted of a 200 μm stripped optical fiber (FT200EMT, Thorlabs) and a twisted pair of teflon-insulated tungsten wires (50 μm uncoated, 112 μm coated; WT-2T, Science Products). The recording electrode protruded ~100 μm with respect to the fiber tip and reference electrode. Another set of electrodes was implanted in the PrC (coordinates from bregma: AP −3.0 mm, ML +4.1 mm, DV 3.8 mm). The optical fiber was coupled to a 80 mW, 470 nm DPSS laser module and the laser output power was attenuated with a neutral density filter wheel (NDM4/M, Thorlabs) and a variable multimode attenuator (MM-ATN-FC, Precision Fiber Products). To exclude a possible crosstalk between the recording sites, each pair of electrodes (recording and reference electrode in area CA1 and the PrC) was connected to an individual headstage. Band-pass filtered data (0.1–500 Hz) was acquired at 2.5 kHz using the Open-Ephys system (Voigts et al., 2013;^1^). After termination of the experiment, electrical lesions were set at recording sites (3 μA for 3 min, negative polarity). Each brain was then cut into 50 μm sections and stained (standard cresyl violet staining) to verify the position of each electrode. Estimation of illumination spots was conducted with an online calculator^2^.

### Chemicals

Atropine sulfate, bicuculline methiodide (BIM), methylenecyclopropylglycine (MCPG), tetrodotoxin (TTX), and all compounds for the saline/intracellular solutions were purchased from Sigma-Aldrich. (2R)-amino-5-phosphonovaleric acid (APV) and 2,3-dihydroxy-6-nitro-7-sulfamoyl-benzo[f]quinoxaline-2,3-dione (NBQX) were from Ascent Scientific, human/rat corticotropin-releasing factor (CRF) from Bachem, and isoflurane from Abbott. Drugs were bath applied to slices. APV, atropine, BIM, CRF, MCPG, NBQX, and TTX were always used at final concentrations of 50, 50, 10, 0.125, 100, 5, and 1 μM, respectively.

### Data Analysis and Statistics

Autocorrelation and spectral analyses (fast Fourier transformations) were conducted with the Igor Pro software. Spectrogram representations were performed using MATLAB Chronux toolbox. Statistical analysis was run in SigmaStat, with significance declared at *p* < 0.05. Data are given as mean ± SEM.

## Results

### Control Experiments in Brain Slices from NEX-Cre-ChR2 Mice

**Figure 1:**
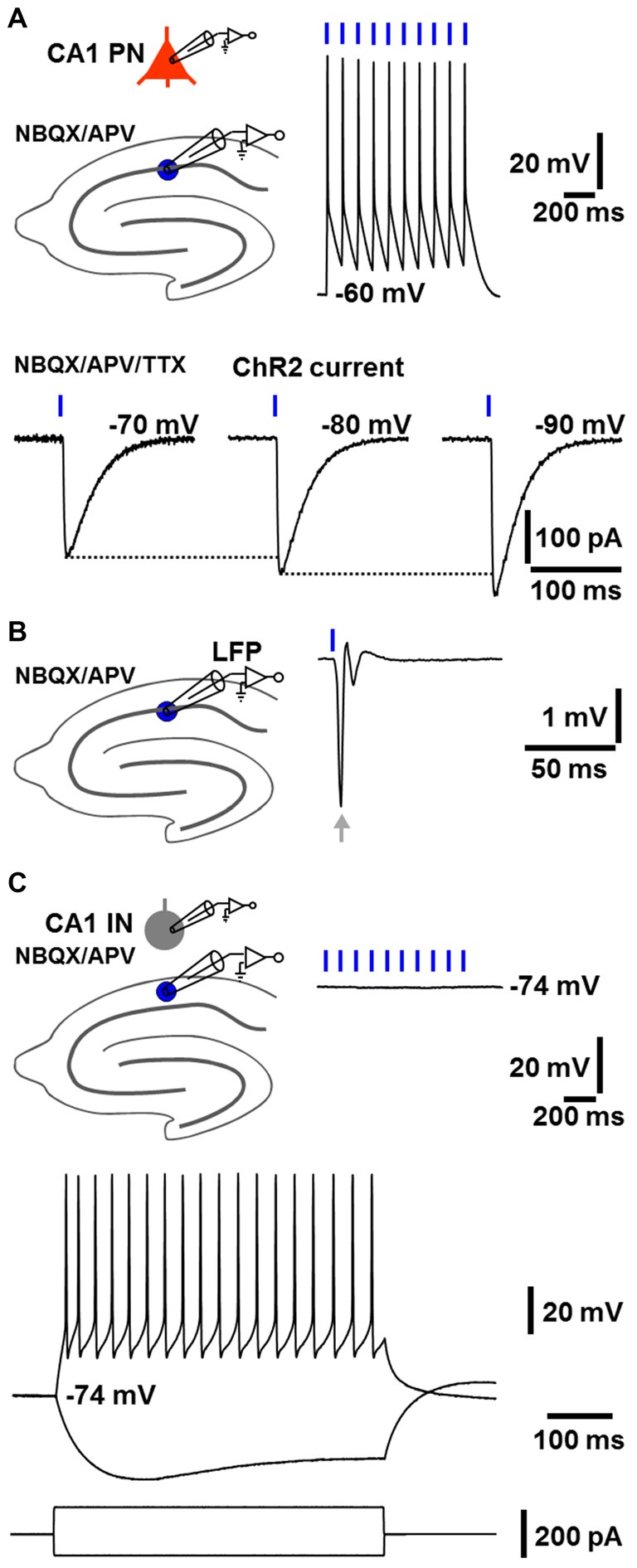
**NEX-Cre-Channelrhodopsin-2 (ChR2) mice express functional ChR2 selectively in glutamatergic cells. (A)** As measured by whole-cell patch-clamp recordings under blockade of ionotropic glutamate receptors by 2,3-dihydroxy-6-nitro-7-sulfamoyl-benzo[f]quinoxaline-2,3-dione (NBQX) and (2R)-amino-5-phosphonovaleric acid (APV), CA1 pyramidal neurons (CA1 PNs) reliably fired action potentials in response to blue light pulses (2 ms). Consequently, ChR2 currents, which increased with more negative membrane potentials, could be detected in CA1 PNs (extracellular solution additionally contained the sodium channel blocker tetrodotoxin, TTX). **(B)** As revealed by local field potential (LFP) recordings in the CA1 stratum pyramidale, a single light pulse invariably triggered a prominent population spike generated by CA1 PNs (arrow, extracellular solution contained NBQX and APV). **(C)** CA1 stratum oriens interneurons (CA1 INs), which exhibited firing characteristics of fast-spiking interneurons upon positive current injection, showed no membrane potential deflections in response to blue light pulses (extracellular solution contained NBQX and APV).

First, we tested in dorsal hippocampal slices whether our self-generated NEX-Cre-ChR2 mice express functional ChR2 selectively in glutamatergic cells. Indeed, as revealed by whole-cell patch-clamp recordings under blockade of ionotropic glutamate receptors by NBQX (5 μM) and APV (50 μM), CA1 pyramidal neurons (*n* = 6 from 4 animals; resting membrane potential (RMP): −62 ± 0.8 mV) reliably fired action potentials in response to blue laser light pulses (488 nm; 2 ms; 4 mW/mm^2^; diameter of light spot: ~150 μm). Consistently, ChR2 currents could be detected in these cells (*n* = 3 from 3 animals), which increased with more negative membrane potentials (Boyden et al., 2005; extracellular solution additionally contained the sodium channel blocker TTX (1 μM); Figure 1A). ChR2 currents in CA1 pyramidal neurons could also be observed in other experiments (*n* = 3), which are described in the third paragraph of the “Results” section. Furthermore, as measured by LFP recordings in the CA1 stratum pyramidale, a single light pulse invariably evoked a prominent population spike generated by pyramidal neurons (*n* = 7 from 5 animals, Figure 1B, extracellular solution contained NBQX and APV). In contrast, CA1 stratum oriens interneurons (*n* = 3 from 3 animals; RMP: ≤−74 mV), which displayed firing characteristics of fast-spiking interneurons upon positive current injection, never showed RMP deflections in response to light pulses (Figure 1C, extracellular solution contained NBQX and APV). The statistical strength of this finding is increased by further patch-clamp recordings from CA1 stratum oriens interneurons (*n* = 3), where the cells likewise failed to exhibit RMP deflections in the presence of NBQX and APV if exposed to ChR2-activating light (see third paragraph of the “Results” section).

### Local Optogenetic Induction of Fast CA1 Network Oscillations in Brain Slices from NEX-Cre-ChR2 Mice: Descriptive Properties, Pharmacological Characteristics, and Impairment by CRF

**Figure 2:**
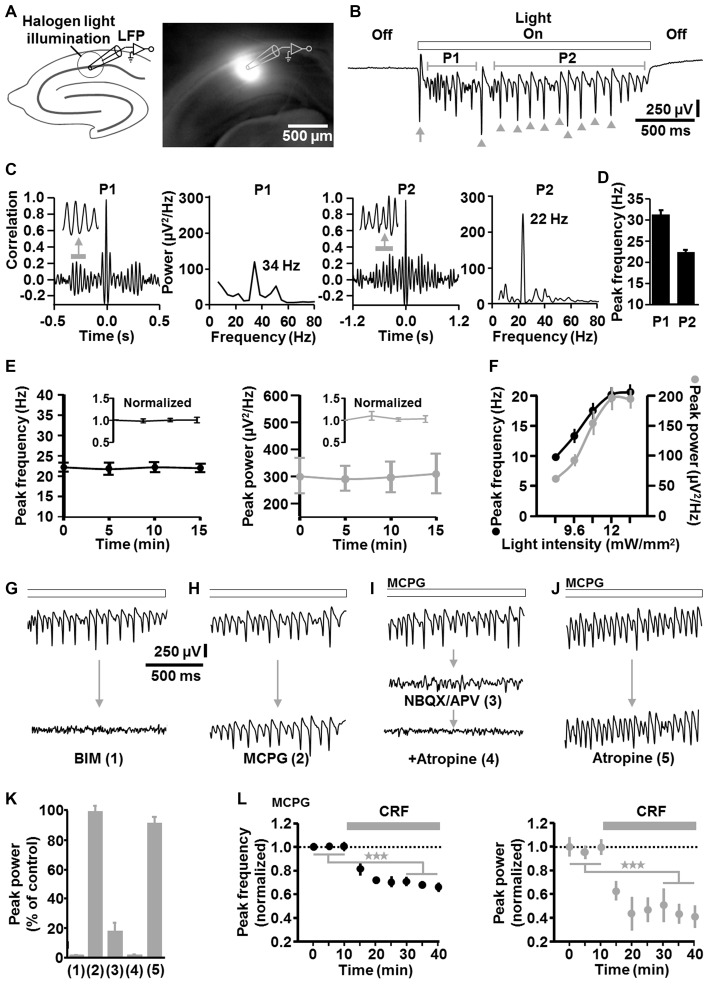
**Descriptive properties and pharmacological characteristics of optogenetically induced fast CA1 network oscillations in brain slices from NEX-Cre-ChR2 mice. (A)** Experimental setting and photograph illustrating the illumination of a circular portion of area CA1 with halogen light (12 mW/mm^2^). **(B)** Representative recording trace depicting the oscillatory network activity during ChR2 activation (arrow and arrow heads mark(s) clearly visible initial and subsequent population spike(s) generated by CA1 pyramidal neurons; P1, period 1; P2, period 2). **(C)** Autocorrelograms and power spectra for P1 and P2 of the rhythmic network activity shown in **(B)**. **(D)** Average data from spectral analyses (*n* = 14 from 7 animals). **(E)** The peak frequency and peak power of the CA1 oscillations remained stable over repeated evocations (*n* = 8 from 4 animals). **(F)** Relationship between halogen light intensity and peak frequency/peak power of the CA1 oscillations (*n* = 8 from 6 animals). Light intensities above 12 mW/mm^2^ did not further increase both parameters of the oscillations. Note the increase of the frequency with increasing spectral power, which is typical for P-I oscillations. **(G–K)** Impact of the GABA_A_ receptor antagonist bicuculline methiodide (BIM; *n* = 7 from 5 animals), the group I/II mGlu receptor blocker methylenecyclopropylglycine (MCPG; *n* = 3 from 3 animals), the ionotropic glutamate receptor antagonists NBQX and APV (*n* = 5 from 4 animals), and the mACh receptor blocker atropine (*n* = 4 from 3 animals for atropine alone) on the light-induced oscillations (all receptor blockers were administered at well proven maximal antagonizing concentrations). For each pharmacological condition, recordings were made 10 min after addition of the drug(s) to the extracellular solution and representative recording traces are shown in **(G–J)**. Quantifications of the pharmacological effects are graphed in **(K)**, with numbers 1–5 corresponding to the numbers in brackets in **(G–J)**. **(I,J)** Extracellular solution contained MCPG. **(L)** Corticotropin-releasing factor (CRF) impaired the light-evoked CA1 oscillations. After recording a stable baseline, human/rat CRF was bath applied at 125 nM to slices. This treatment led to a marked decrease in the peak frequency and peak power of the oscillations (*n* = 7 from 4 animals, ^★★★^*p* < 0.001, one-sample *t* test). Extracellular solution contained MCPG.

Next, we conducted LFP recordings in the CA1 stratum pyramidale *in vitro* and directed halogen light (from a stabilized light source) via the condenser of an upright microscope onto a circular portion of area CA1 (~300 μm diameter), which enclosed the stratum oriens, pyramidale, and radiatum (Figure 2A). ChR2 activation by halogen light is enabled by the overlap of the halogen light and ChR2 activation spectrum (Zhang et al., 2010) and was used to achieve a spatially accurate ChR2 activation (and its rapid fine adjustment) in the defined circular area of the CA1 region. The diameter of the illumination spot could be quickly and steplessly changed by altering the opening of the condenser diaphragm. A temporally precise activation of ChR2 was realized by means of a shutter that was positioned between the light source and the condenser. For comparability with visually guided patch-clamp recordings (see below), experiments were carried out in a submerged-type slice chamber. According to the studies of Mann et al. (2005) and Lu et al. (2012), measurements were performed at sub-physiological temperature (i.e., 25°C in the present work; Section “Discussion”).

Sustained light application (2 s, 12 mW/mm^2^) reliably evoked oscillatory-like activity, which accurately followed the “light off-on-off” cycle. The appearance and the magnitude of the voltage deflections of this activity strongly resemble that of several forms of experimentally induced CA1 or CA3 gamma oscillations recorded in the stratum pyramidale *in vitro* (e.g., Fellous and Sejnowski, 2000; Akam et al., 2012; Tsintsadze et al., 2015; Figure 2B). Autocorrelations and power spectra confirmed that the oscillatory-like activity represents network oscillations and revealed mean peak frequencies of 31.2 ± 1.3 Hz for milliseconds 100–600 (“period 1”) and 22.3 ± 0.8 Hz for milliseconds 800–2000 (“period 2”) of light application (*n* = 14 from 7 animals; Figures 2C,D). Hence, the oscillations in period 1 are in the classical gamma frequency range, but somewhat below in period 2. However, gamma oscillations in hippocampal slices have been repeatedly reported to show frequencies of ~20 Hz if measured at ~25°C (as done here), but to fall clearly in the classical gamma band when recorded at or above 32°C (Dickinson et al., 2003; Mann et al., 2005; Lu et al., 2012). Nevertheless, for correctness, we term the light-induced oscillations “fast CA1 network oscillations” at some locations of this manuscript. As shown in Figure 2B (arrow heads), the optogenetically triggered oscillations mostly comprised clearly visible population spikes, suggesting an involvement of rhythmic synchronous firing of pyramidal neurons. Such ensemble activity is characteristic for P-I oscillations (ter Wal and Sejnowski, 2014). Due to their higher stability and spectral power in period 2 (Figure 2C), all following analyses of the oscillations were done for this time frame.

The CA1 oscillations remained stable over repeated evocations (*n* = 8 from 4 animals, Figure 2E). Up to the aforementioned frequency of ~22 Hz in period 2, both the frequency and spectral power of the rhythmic network activity increased with increasing light intensities (*n* = 8 from 6 animals, Figure 2F). Thus, the frequency increased with increasing spectral power, representing a relationship that is typical for P-I oscillations (ter Wal and Sejnowski, 2014). Such a relationship was likewise observed for the oscillatory activity in period 1 (data not shown).

Corroborating a critical role for inhibitory interneurons (Bartos et al., 2007), GABA_A_ receptor blockade by BIM (10 μM) disrupted the rhythmic CA1 activity (*n* = 7 from 5 animals; Figures 2G,K). In contrast, group I/II metabotropic glutamate (mGlu) receptor antagonism by MCPG (100 μM) did not distinctly affect the oscillations (*n* = 3 from 3 animals; Figures 2H,K), indicating no or only a minor contribution of interneuron network rhythmicity (Whittington et al., 1995; Tiesinga and Sejnowski, 2009; note the nearly complete erasement of CA1 interneuron network oscillations by MCPG in Whittington et al., 1995). Nevertheless, to eliminate any interneuron network oscillations, all further *in vitro* experiments were conducted under continuous group I/II mGlu receptor inhibition. Consistent with the three measurements where no clear MCPG effects could be detected (Figures 2H,K), halogen light application caused CA1 oscillations in these experiments, which were comparable to those induced in the absence of MCPG (Figures 2G–J, upper traces). Blockade of ionotropic glutamate receptors by NBQX (5 μM) and APV (50 μM) almost completely abrogated the oscillations (*n* = 5 from 4 animals; Figure 2I (middle trace), Figure 2K). Interestingly, the small remaining component was abolished by the muscarinic acetylcholine (mACh) receptor antagonist atropine (50 μM; Figure 2I (lower trace), Figure 2K; Section “Discussion”). However, inhibition of mACh receptors alone did not markedly impact the oscillations (*n* = 4 from 3 animals; Figures 2J,K), demonstrating that activation of these receptors is not required for the generation of the rhythmic network activity.

To test whether the light-evoked oscillations are also sensitive to endogenous neuromodulators, we applied CRF (125 nM) to slices. Stress hormones like CRF can interfere with hippocampus-dependent cognitive functions and gamma frequency activity in CA regions (e.g., Radulovic et al., 1999; Çalişkan et al., 2015), but direct effects of CRF on fast CA1 network oscillations have not been probed to date. Interestingly, while CRF facilitates ACh-induced gamma rhythmicity in area CA3 (Çalişkan et al., 2015), we observed a marked decrease in the peak frequency and peak power of the CA1 oscillations (*n* = 7 from 4 animals, Figure 2L).

### Single-Cell Recordings in Brain Slices from NEX-Cre-ChR2 Mice

**Figure 3:**
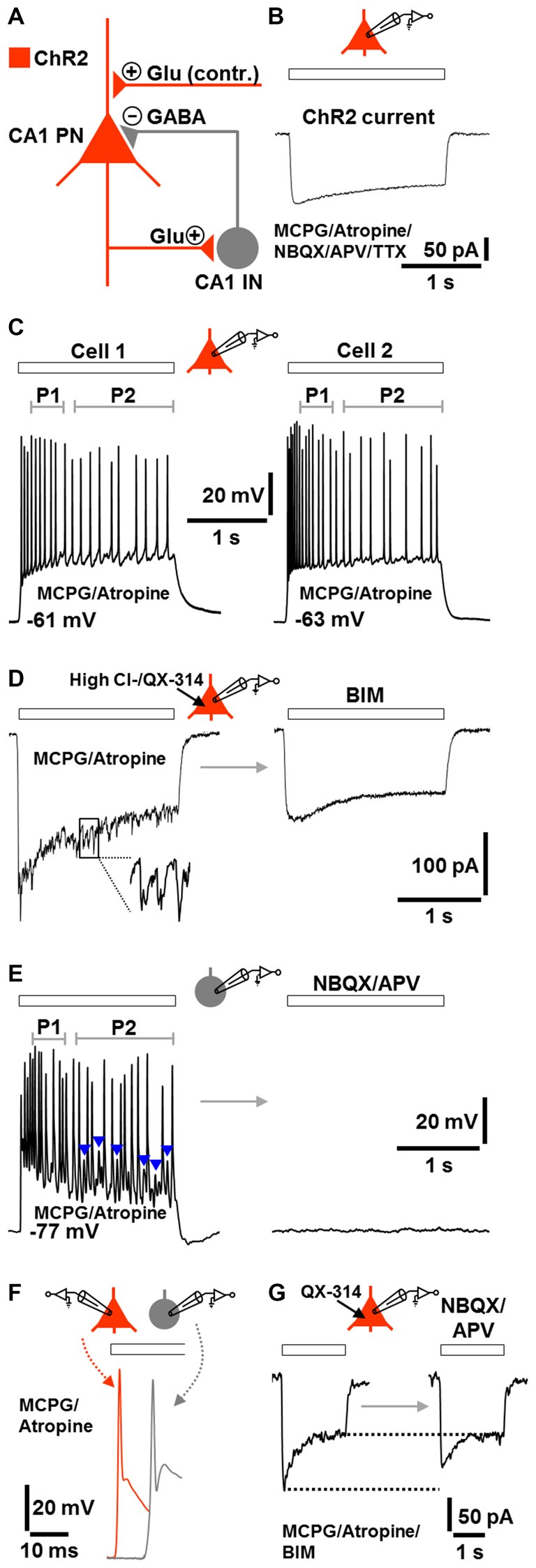
**Model of the network components and signaling modalities underlying or contributing (contr.) to the optogenetically induced fast CA1 oscillations under group I/II mGlu/mACh receptor blockade (A) and corroborative single-cell activities during ChR2 activation (B–G; Glu, glutamate).** All recordings were performed in the continuous presence of MCPG and atropine. If not stated otherwise, recordings were made using a K-gluconate-based intracellular solution. Halogen light intensity was always 12 mW/mm^2^. Holding potential in voltage-clamp measurements was −70 mV. Application time of drug(s) in **(D,E,G)** was 10 min. **(B)** ChR2 current in a CA1 PN evoked by halogen light illumination (extracellular solution additionally contained NBQX, APV, and TTX). **(C)** CA1 PNs immediately fired action potentials in response to halogen light illumination and continued spiking until the end of light application. The frequency of action potentials in P2 was lower than in P1. Representative recording traces from two cells are shown. **(D)** Fast postsynaptic inward currents in a CA1 PN during halogen light delivery. These currents could be unmasked by recordings with a high Cl^−^ intracellular solution containing the sodium channel blocker QX-314. The currents promptly subsided when light delivery was terminated and were completely blocked by the GABA_A_ receptor antagonist BIM. **(E)** Whole-cell patch-clamp recording from a CA1 stratum oriens IN. Although lacking ChR2, the neuron continuously fired during halogen light application and spike trains were interspersed with prominent excitatory postsynaptic potentials (EPSPs; blue arrow heads). As observed for CA1 PNs, the frequency of action potentials in P2 was lower than in P1. The cell was totally silenced by ionotropic glutamate receptor blockade by NBQX and APV. **(F)** Illustration of the time delay (~10 ms) between the first action potential in CA1 INs and the first action potential in CA1 PNs. Representative recording traces from separate measurements are shown. **(G)** Light-evoked inward currents in a CA1 PN (in the presence of BIM) before and after addition of NBQX and APV to the extracellular solution (intracellular solution contained QX-314). Representative recording traces are depicted.

The fast CA1 network oscillations under group I/II mGlu/mACh receptor blockade (Figure 2J, lower trace) most likely represent prototypic P-I oscillations. A putative circuit model, which considers the possibility that ChR2 activation caused transmitter release at glutamatergic synapses (e.g., Schaffer collateral synapses) onto CA1 pyramidal neurons (Felix-Ortiz et al., 2013), is depicted in Figure 3A. To test the validity of this model, we performed whole-cell patch-clamp recordings from CA1 pyramidal neurons, which were located in the region of halogen light illumination. These cells exhibited moderate ChR2 currents (100–150 pA) with typical desensitization kinetics (Boyden et al., 2005) in response to halogen light (12 mW/mm^2^; *n* = 3 from 3 animals; Figure 3B; cf., Figure 1A). We also recorded from CA1 stratum oriens interneurons, which predominantly mediate feedback inhibition of CA1 pyramidal cells (e.g., Blasco-Ibáñez and Freund, 1995). Recordings were conducted under group I/II mGlu/mACh receptor inhibition and, if not stated otherwise, made using a K-gluconate-based intracellular solution. The results obtained confirm the model and are as follows.

The pyramidal neurons and interneurons did not discharge in the absence of ChR2 activation. When the light was switched on, all pyramidal neurons immediately responded with action potentials and continued spiking until the end of light application (*n* = 7 from 5 animals). Probably due to spike frequency adaptation (Madison and Nicoll, 1984), the rate of action potentials in period 2 was lower than in period 1 (Figure 3C). This phenomenon is likely to underlie, at least in part, the lower frequency of the CA1 network oscillations in period 2 if compared to period 1 (Figure 1D).

In another set of experiments, we recorded from pyramidal neurons using a high Cl^−^ intracellular solution containing the sodium channel blocker QX-314. Under these conditions, we registered fast postsynaptic inward currents during the whole time of light application in all measurements conducted. Invariably, these currents promptly vanished when light delivery was terminated and were completely blocked by the GABA_A_ receptor antagonist BIM (*n* = 3 from 3 animals, Figure 3D).

Although lacking ChR2, three of the four interneurons recorded also continuously fired during light application and spike trains were interspersed with prominent excitatory postsynaptic potentials (EPSPs). As observed for pyramidal cells, the frequency of action potentials in period 2 was lower than in period 1. The interneurons (RMP: ≤−76 mV), which showed fast-spiking discharge characteristics upon positive current injection (data not shown, but see Figure 1C), were totally silenced by ionotropic glutamate receptor blockade (cells were from 3 animals, Figure 3E). When compared to pyramidal cells, the first action potential generated in the interneurons appeared with a delay of ~10 ms (Figure 3F). Furthermore, under GABA_A_ receptor blockade, light-evoked inward currents in pyramidal neurons were diminished by ionotropic glutamate receptor inhibition (*n* = 3 from 2 animals, Figure 3G), confirming that ChR2 activation caused transmitter release at glutamatergic synapses onto pyramidal neurons. However, the reduction of the currents was weak and restricted to their initial phase, indicating that this transmitter release is not essential for the optogenetically triggered oscillations.

### Local Optogenetic Induction of Fast CA1 Network Oscillations in Slightly Anesthetized NEX-Cre-ChR2 Mice

**Figure 4:**
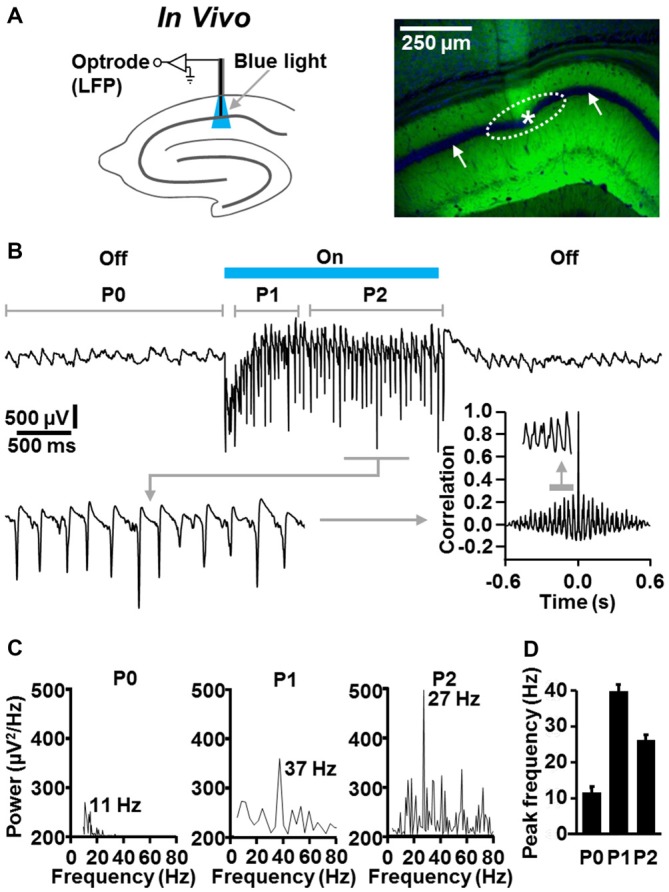
**Temporally precise optogenetic induction and termination of fast CA1 network oscillations in slightly anesthetized NEX-Cre-ChR2 mice. (A)** Experimental setting and photograph illustrating the position of the recording tip of the optrode (asterisk) and the calculated illumination spot in the CA1 stratum pyramidale (dotted ellipse) in the experiment shown in (**B**; right panel: green (EYFP-generated) fluorescence signal reports ChR2 expression; blue fluorescence signal reports nuclear DAPI staining, which is best visible in the pyramidal cell body layer (white arrows)). **(B)** Representative recording trace, enlarged portion of the trace, and autocorrelogram depicting the oscillatory network activity in the absence and presence of ChR2 activation by blue laser light (488 nm; 4 mW/mm^2^). **(C)** Power spectra for P0, P1, and P2 of the rhythmic network activity shown in **(B)**. **(D)** Average data from spectral analyses (*n* = 5).

Next, we examined whether fast CA1 network oscillations likewise can be optogenetically evoked under *in vivo* conditions. For this purpose, we performed optrode LFP recordings (Zhang et al., 2010) in the left dorsal CA1 stratum pyramidale of slightly isoflurane-anesthetized NEX-Cre-ChR2 mice (Figure 4A). Although it has been shown that general anesthetics, including isoflurane, can slow hippocampal gamma oscillations (e.g., Dickinson et al., 2003), we chose this proceeding to reduce the probability of seizure generation.

In the absence of ChR2 activation, we detected ongoing theta activity in all animals tested (*n* = 5; Figures 4B–D; mean peak frequency in period 0: 10.7 ± 0.9 Hz). Much faster and stronger CA1 network oscillations could be evoked instantaneously by ChR2 activation by blue laser light (488 nm; 4 mW/mm^2^; estimated diameter of illumination spot in stratum pyramidale: ~300 μm). In period 1, these oscillations were in the classical gamma frequency range (39 ± 1.7 Hz), but somewhat below in period 2 (26.2 ± 0.9 Hz). The optogenetically induced oscillatory activity was never accompanied by seizures in the experimental animals and promptly subsided when light delivery was terminated (Figures 4B–D). As observed in the *in vitro* experiments (Figure 2B), the fast CA1 oscillations comprised rhythmically occurring population spikes. In line with the presence of P-I oscillations, the rate of the population spikes matched the frequency of the oscillations (ter Wal and Sejnowski, 2014; Figure 4B, enlarged trace).

### Light-Evoked Fast CA1 Network Oscillations in NEX-Cre-ChR2 Mice are Accompanied by Concurrent States of Enforced Perirhinal Theta Activity

**Figure 5:**
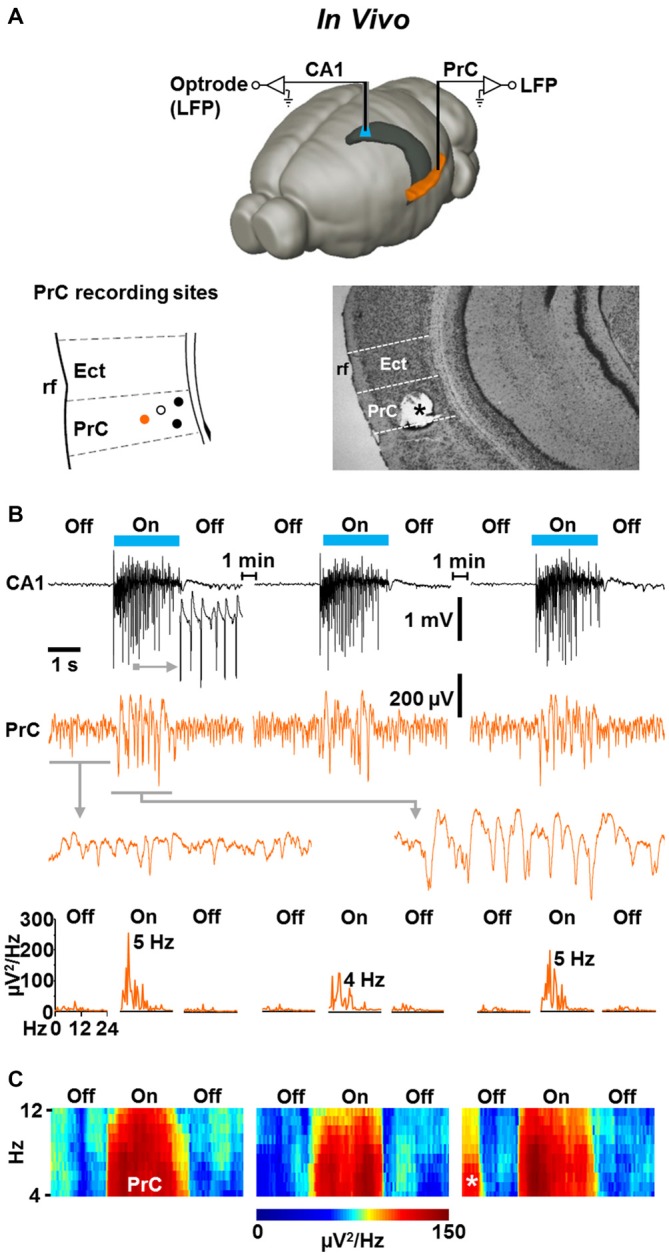
**Light-induced fast CA1 network oscillations in NEX-Cre-ChR2 mice are accompanied by concurrent states of enforced theta activity in the ipsilateral PrC. (A)** Experimental setting (upper panel) and schematic illustration of PrC recording sites in a part of a coronal brain section (lower left panel; Ect, ectorhinal cortex; rf, rhinal fissure). Closed circles represent recording sites where enforced theta activity was detected. Closed orange circle indicates the recording site in the experiment shown in **(B)**. Open circle marks recording site at which no enforced theta activity was detected. Lower right panel: for verification of PrC recording sites, electrical lesions were set after electrophysiological measurements. Asterisk marks the position of the recording electrode in the experiment shown in **(B)**. **(B)** Representative recording traces (and enlarged portions of the traces) depicting the CA1 and simultaneous PrC network activities during the repetitive “light off-on-off” protocol used. Lower panels give power spectra for the PrC measurements. **(C)** Average spectrograms for the PrC recordings marked by closed circles in the lower left panel in **(A)**. Asterisk indicates prominent spontaneous theta activity.

Finally, we addressed the question of whether the light-induced CA1 oscillations impact neuronal activity in the PrC *in vivo*. We performed simultaneous optrode and classical LFP recordings in the left dorsal CA1 stratum pyramidale and ipsilateral caudoventral PrC (van Groen and Wyss, 1990) of slightly isoflurane-anesthetized NEX-Cre-ChR2 mice (Figure 5A, upper panel). In all experiments in which the recording electrode was on target in the PrC (Figure 5A, lower panels), we registered weak, but clearly visible theta activity (Collins et al., 1999) in the absence of ChR2 activation (Figures 5B,C; *n* = 4; mean peak frequency and peak power before “light on”: 7.1 ± 0.9 Hz and 41 ± 6 μV^2^/Hz, respectively). Remarkably, when the light was switched on, the spectral power of this rhythmic activity rapidly and strongly increased in three out of the four experiments conducted, while the frequency did not significantly change (mean peak frequency and peak power during “light on”: 6.3 ± 1.1 Hz and 143 ± 19 μV^2^/Hz, respectively). This effect lasted for the whole time of light application, instantaneously disappeared when the CA1 oscillations were terminated, and, increasing statistical strength, could reliably be induced in a repetitive manner (repetitive “light off-on-off” procedure; Figures 5B,C).

## Discussion

In the present study, we investigated whether dorsal area CA1 of mice harbors the circuitry for a generation of prototypic P-I gamma oscillations and, if so, whether such activity can impact network oscillations in the memory-associated PrC (Zola-Morgan et al., 1993; Bilkey and Heinemann, 1999; Fell et al., 2001). For this purpose, we performed different types of electrophysiological measurements in NEX-Cre-ChR2 mice, which selectively express the excitatory opsin ChR2 in forebrain glutamatergic cells (and thus in CA1 pyramidal neurons), but not in GABAergic interneurons. By means of this optogenetic approach, we were able to locally, temporally precisely, and repetitively induce fast (20–40 Hz) CA1 network oscillations in acute brain slices (at 25°C) and slightly isoflurane-anesthetized animals. As revealed by pharmacological analyses and patch-clamp recordings from pyramidal cells and stratum oriens interneurons *in vitro*, these light-triggered oscillations can exclusively arise from sustained suprathreshold depolarization (~200 ms or longer) and feedback inhibition of pyramidal neurons, as being mandatory for prototypic P-I oscillations (Bartos et al., 2007; Tiesinga and Sejnowski, 2009; ter Wal and Sejnowski, 2014). Consistently, the oscillations comprised rhythmically occurring population spikes (generated by pyramidal cells) during their whole lifetime and their frequency increased with increasing spectral power, representing a relationship that is typical for P-I oscillations (ter Wal and Sejnowski, 2014).

We found that during milliseconds 100–600 (period 1) of sustained ChR2 activation, the optogenetically evoked CA1 oscillations were in the classical gamma frequency range (~31 Hz *in vitro* and ~39 Hz *in vivo*), but somewhat below during milliseconds 800–2000 (period 2, ~22 Hz *in vitro* and ~26 Hz *in vivo*). While this slowing probably originates, at least in part, from spike frequency adaptation of pyramidal neurons (Figure 3C), the relatively low or outlying frequency of the oscillatory activity with respect to the classical gamma band most likely results from the temperature at which *in vitro* experiments were conducted (25°C) and the use of isoflurane-anesthetized animals for *in vivo* measurements (Dickinson et al., 2003; Mann et al., 2005; Lu et al., 2012). However, according to the data from Lu et al. (2012), we decided to perform slice recordings at 25°C, in order to assure a good oxygen and mitochondrial ATP supply of neurons under submerged conditions and to still achieve a relatively high spectral power of potentially occurring oscillations. *In vivo* experimentation in isoflurane-anesthetized mice was done to reduce the probability of seizure generation. Given the findings that the frequency of carbachol-evoked CA1 and kainate-induced CA3 gamma oscillations in hippocampal slices increases by 2–3 Hz in response to a 1°C elevation of the experimental temperature (in the range of 23–34°C; Dickinson et al., 2003; Lu et al., 2012), one can expect that the frequency of our light-triggered CA1 oscillations *in vitro* would be considerably higher (and invariably in the classical gamma band) at temperatures of ~30–32°C or above. With respect to the *in vivo* oscillations, a similar scenario appears likely for anesthesia-free conditions, since general anesthetics, including isoflurane, can markedly slow hippocampal gamma activity (e.g., Dickinson et al., 2003).

Interestingly, ChR2 activation gave rise to weak ACh-driven oscillatory activity (Figures 2I,K). Possible explanations for this observation are that light-triggered glutamate release excited “cholinergic” interneurons (Yi et al., 2015) and/or that glutamate/ACh co-releasing fibers (Allen et al., 2006), putatively endowed with ChR2, were activated.

The optogenetically induced CA1 oscillations *in vitro* remained stable over repeated evocations, making them usable for pharmacological investigations. Indeed, in a side project of this study, we could show that the stress hormone CRF (125 nM) decreases the peak frequency and peak power of the rhythmic network activity. While the detailed mechanisms underlying this effect are currently unknown, their elucidation may be a worthwhile topic for future work, given that CRF has been reported to facilitate ACh-induced gamma oscillations in area CA3 (Çalişkan et al., 2015).

A remarkable result of our work is that the locally light-triggered CA1 oscillations in NEX-Cre-ChR2 mice are accompanied by concurrent states of enforced theta activity in the ipsilateral PrC. While the detailed mechanisms of this repetitively inducible phenomenon remain to be clarified, its immediate appearance/disappearance after starting/terminating the CA1 oscillations strongly suggest a major role for excitatory synaptic inputs from CA1 and/or subicular pyramidal cells to PrC neurons (Kealy and Commins, 2011), rather than an indirect effect via the septum. Consistent with this scenario, layer III/V PrC neurons instantaneously show intrinsic theta frequency membrane potential oscillations and theta-modulated firing if depolarized to ~−50 mV or higher (Bilkey and Heinemann, 1999). It is likely that gamma or slightly sub-gamma frequency EPSPs in these cells, which putatively occurred during the optogenetically evoked CA1 oscillations, undergo summation and, thus, can produce such depolarizations. However, one has also to consider that area CA1 receives glutamatergic projections from the PrC (van Groen and Wyss, 1990), which probably carry ChR2 in NEX-Cre-ChR2 mice. Yet, it is safe to assume that the sustained light application to area CA1 triggered, if at all, only a single antidromic spike in these fibers (Tye et al., 2011), while, due to their length of ~5–6 mm (according to the Allen brain atlas), the residual potential change underwent severe electrotonic attenuation (Debanne et al., 2011). We therefore exclude light-induced axonal depolarizations of PrC neurons as a significant driving force for the observed up-shifts of perirhinal theta activity power.

Finally, it is important to mention that a recent study used selective optogenetic activation of CA1 pyramidal neurons to evoke CA1 ripple (>90 Hz) oscillations in freely moving rats (Stark et al., 2014). The seeming discrepancy to our work probably relies on partly significant methodological differences. For instance, the authors used much shorter epochs of light application (50–100 ms), employed considerably lower blue light intensities (~0.1–0.2 mW/mm^2^) for ChR2 activation, and illuminated a smaller area of the CA1 region than we did. Given the assumed critical role of appropriate magnitudes of convergent excitatory drive from pyramidal cells on interneurons and divergent inhibitory drive from interneurons on pyramidal cells for the generation of P-I gamma oscillations in the hippocampus (e.g., ter Wal and Sejnowski, 2014), it is possible that Stark et al. (2014) exclusively induced ripple oscillations, while, in our experiments, the equivalent of this high-frequency oscillatory activity is superimposed by slower, but more powerful P-I oscillations. Consistent with this scenario, the power spectra shown in Figures 2C, 4C exhibit submaximal peaks in the range of 40–60 Hz, which might represent ripple oscillations in higher temperature *in vitro* measurements or anesthesia-free *in vivo* recordings. However, there are also analogies to our study. In particular, Stark et al. (2014) revealed that the optogenetically triggered ripple oscillations involve GABAergic interneuron activity and found that pyramidal cell spiking precedes interneuron spiking during this process.

In summary, our data provide evidence for the existence of a prototypic (CRF-sensitive) P-I gamma rhythm generator in area CA1 and suggest that CA1 P-I oscillations can rapidly up-regulate theta activity strength in hippocampus-innervated rhinal networks, at least in the PrC.

## Author Contributions

JD and AG designed, performed, and analyzed experiments. FH analyzed data. CTW, JMD, WZ, and AC designed experiments and/or provided materials. ME designed and analyzed experiments, coordinated the study, and wrote the manuscript.

## Conflict of Interest Statement

The authors declare that the research was conducted in the absence of any commercial or financial relationships that could be construed as a potential conflict of interest.

## Footnotes

^1^http://www.open-ephys.org/

^2^http://web.stanford.edu/group/dlab/cgi-bin/graph/chart.php
