# Corrigendum: Local Optogenetic Induction of Fast (20–40 Hz) Pyramidal-Interneuron Network Oscillations in the *In Vitro* and *In Vivo* CA1 Hippocampus: Modulation by CRF and Enforcement of Perirhinal Theta Activity

**DOI:** 10.3389/fncel.2016.00148

**Published:** 2016-06-10

**Authors:** Julien Dine, Andreas Genewsky, Florian Hladky, Carsten T. Wotjak, Jan M. Deussing, Walter Zieglgänsberger, Alon Chen, Matthias Eder

**Affiliations:** ^1^Max Planck Institute of PsychiatryMunich, Germany; ^2^Department “Stress Neurobiology and Neurogenetics”, Max Planck Institute of PsychiatryMunich, Germany; ^3^Scientific Core Unit “Electrophysiology and Neuronal Network Dynamics”, Max Planck Institute of PsychiatryMunich, Germany; ^4^Research Group “Neuronal Plasticity”, Max Planck Institute of PsychiatryMunich, Germany; ^5^Research Group “Molecular Neurogenetics”, Max Planck Institute of PsychiatryMunich, Germany; ^6^The Ruhman Family Laboratory for Research on the Neurobiology of Stress, Department of Neurobiology, Weizmann Institute of ScienceRehovot, Israel

**Keywords:** CA1, CRF, gamma, hippocampus, optogenetics, perirhinal cortex, pyramidal-interneuron network oscillations, theta

Corrigendum on: ter Wal, M., and Sejnowski, T. J. (2014). “Hippocampal oscillations, mechanisms (PING, ING, Sparse),” in *Encyclopedia of Computational Neuroscience*, eds D. Jaeger and R. Jung (New York: Springer), 1–14 (p. 11 of the published manuscript, in the references list).

Reason for Corrigendum:

There was a mistake in the author name in the citation mentioned above as published. The correct version of the citation appears below. The authors apologize for the mistake. This error does not change the scientific conclusions of the article in any way.

The correct citation for this publication is “ter Wal, M., and Tiesinga, P. (2014). “Hippocampal oscillations, mechanisms (PING, ING, Sparse),” in *Encyclopedia of Computational Neuroscience*, eds D. Jaeger and R. Jung (New York: Springer), 1–14.”

## Author contributions

JD and AG designed, performed, and analyzed experiments. FH analyzed data. CW, JD, WZ, and AC designed experiments and/or provided materials. ME designed and analyzed experiments, coordinated the study, and wrote the manuscript.

### Conflict of interest statement

The authors declare that the research was conducted in the absence of any commercial or financial relationships that could be construed as a potential conflict of interest.

